# The reality of informed consent: empirical studies on patient comprehension—systematic review

**DOI:** 10.1186/s13063-020-04969-w

**Published:** 2021-01-14

**Authors:** Tomasz Pietrzykowski, Katarzyna Smilowska

**Affiliations:** 1 Research Centre for Public Policy and Regulatory Governance, Faculty of Law and Administration, University of Silesia in Katowice, Katowice, Poland; 2 Department of Neurology; Centre of Expertise for Parkinson & Movement Disorders, Radboud University Medical Center, Donders Institute for Brain, Cognition and Behaviour, PO Box 9101, 6500 HB Nijmegen, the Netherlands

**Keywords:** Informed consent, Clinical trials, Medical practice, Law, Ethics, Comprehension, Health care quality, Autonomy

## Abstract

**Background:**

Informed consent is a basic concept of contemporary, autonomy-based medical practice and facilitates a shared decision-making model for relations between physicians and patients. Thus, the extent to which patients can comprehend the consent they grant is essential to the ethical viability of medicine as it is pursued today. However, research on patients’ comprehension of an informed consent’s basic components shows that their level of understanding is limited.

**Methods:**

Systemic searches of the PubMed and Web of Science databases were performed to identify the literature on informed consent, specifically patients’ comprehension of specific informed consent components.

**Results:**

In total, 14 relevant articles were retrieved. In most studies, few clinical trial participants correctly responded to items that examined their awareness of what they consented to. Participants demonstrated the highest level of understanding (over 50%) regarding voluntary participation, blinding (excluding knowledge about investigators’ blinding), and freedom to withdraw at any time. Only a small minority of patients demonstrated comprehension of placebo concepts, randomisation, safety issues, risks, and side effects.

**Conclusions:**

We found that participants’ comprehension of fundamental informed consent components was low, which is worrisome because this lack of understanding undermines an ethical pillar of contemporary clinical trial practice and questions the viability of patients’ full and genuine involvement in a shared medical decision-making process.

## Introduction

Written informed consent (IC) is considered a basic principle of medical practice. It provides information and shares knowledge between the physician and patient and creates a shared-decision-based healthcare plan [1]. In this regard, the IC should implement a principle of autonomy, by which a patient’s right to deliberately decide for herself whether to accept or refuse the offered treatment must be respected [2, 3]. However, patients’ adequate understanding of the provided information is a major limitation.

Within its ethical and legal foundations, the informed consent process is pivotal to supporting ethically sound medical intervention. However, obtaining adequately informed consent from patients is complex because it requires human interactions involving discussion of several elements, such as the patient’s condition and therapeutic options, including risks and benefits, inconveniences, and uncertainties. In this regard, IC must include both a form that patients are required to read and sign, and oral communication to ensure adequate understanding to facilitate voluntary willingness to participate in a clinical trial [4].

Major barriers to adequate IC understanding include the patients’ subjective impression that they are well informed and physicians’ over-confidence in the intelligibility and quality of the information they provide to patients. Nonetheless, the concept of respecting patients’ autonomy in medical research is based on the assumption that the informed consent process actually leads to patients’ full comprehension of what they are consenting to. Unless this assumption is demonstrably true, the ethical viability of the current medical experimentation practice is seriously flawed.

Given that most available studies focused on informed consent obtained for the purpose of clinical trials, we limited our scope to this kind of research practice. However, there is no reason to assume that the level of understanding of informed consent granted by patients in a routine medical practice is significantly higher than that in clinical trials. On the contrary, we find it plausible that patients recruited to clinical trials are relatively better informed and physicians may explain the nature of a research intervention and participation conditions more thoroughly. Therefore, it is improbable that patients’ actual comprehension of consent in standard medical practice is higher than in the relatively better examined conditions of clinical trials, and there are reasons to expect that it is lower. With this reservation, our conclusions may be extended beyond clinical trial conditions to the more general practice of obtaining informed consent in medical practice.

Therefore, we systematically reviewed the available literature on patients’ actual (rather than declared) understanding of what they consented to, with particular interest in questionnaires developed to objectify patients’ understanding of the consent content, rather than their subjective impression on how well informed they were during the consenting process, and whether they were satisfied with the way in which their consent was obtained.

## Methods

We performed a systematic review using the Preferred Reporting Items for Systematic Review and Meta-Analysis (PRISMA) criteria [5]. The electronic search to identify and capture informed consent literature was conducted between October 2019 and January 2020. We queried PubMed and Web of Science databases using the following search terms: “informed consent [mh] AND (comprehension [mh] OR perception [mh] OR knowledge [mh] OR decision making [mh] OR understanding OR communication [mh]) AND (randomised controlled trials as topic [mh] OR clinical trial as topic [mh])”. No year restrictions were applied. To make the search as comprehensive as possible, we used the Boolean operators “AND” and “OR” to link the search terms.

Inclusion criteria were (a) studies assessing comprehension of IC, (b) English-language articles in peer-reviewed academic/scientific journals, (c) full-text articles available electronically, and (d) articles with available questionnaires used to examine the level of patients’ understanding.

Exclusion criteria were (a) studies comparing or evaluating methods of informed consent not related to IC comprehension (defined in inclusion criteria), (b) studies that used intervention to improve patients’ understanding, (c) studies that included patients with cognitive decline, (d) qualitative research, (e) articles based on patients’ impression of understanding, (f) studies based on interviews, (g) studies that did not provide the questionnaire used, (h) conference abstracts, and (i) animal studies.

We included only articles that examined knowledge about the information included in the IC. In this regard, we excluded articles based on interviews and questionnaires that examined only patients’ impressions of understanding (e.g. “Did you receive adequate information about the study?”).

Article selection was performed independently by the first (TP) and second (KS) authors. Database searches were completed in a blinded manner using identical search terms. After identifying eligible articles, any doubts were resolved during a meeting to review the queried article(s) against the inclusion and exclusion criteria. The final selection of eligible articles included in the critical appraisals was made based on the agreement between TP and KS.

## Results

### Selection process

**Fig. 1:**
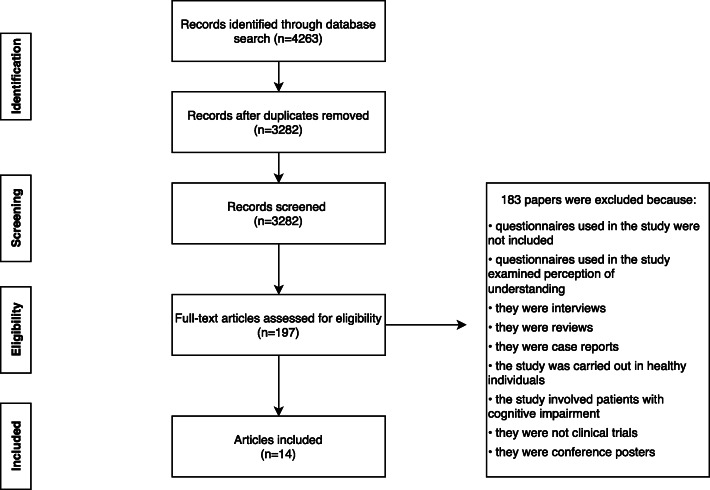
Selection process for eligible articles

The study selection process is shown in Fig. 1. In total, 4263 articles were retrieved from the databases, of which 14 were included in the review based on the inclusion/exclusion criteria (Table 1). The number of participants varied across studies, ranging from 29 [16] to 1835 [9]. In most studies (*n* = 12), participants were adults [7–15, 17–19]; three studies examined parents or guardians [6, 10, 16]; and one study included both adult patients and parents or guardians [10]. Medical specialties included infectious disease in 42% (*n* = 6), including vaccine studies in 21% (*n* = 3), oncology in 28% (*n* = 4); rheumatology in 21% (*n* = 3); neurology in 7% (*n* = 2), and others in 7%. Two studies included clinical trials in more than one specialty [8, 15]. Most studies examined IC-related questions that covered compensation, withdrawal criteria and consequences, study versus treatment, study administration, and randomisation.

**Table 1:** Studies included in the review of patients understanding of informed consent in clinical trials

Author [year]	Country	Number of patients	Age	Subject of the study	Population	Timing	Aspects of IC explored in the study and percentage of correct answers	Level of education
Ponzio [6] [2018]	Italy	60	52.9 ± 11.7	Neurology	Adult patients	ND	Purpose of the study 20.7–93.5% Blindness 58.6–89.7% Freedom to withdrawal 100% Confidentiality 29–93.1% Risk 6.9–100% Benefits 34.5–96.6%	ND
Schumacher [7] [2017]	USA	50	61 (35–85)	Oncology	Adult patients	Mean time 60 ± 51 Days after ICP for treatment (oncology) trial	Risk 20% Direct benefits 76% Freedom to withdrawal 90% Voluntary participation 90%	Less than high school diploma 9% High school diploma 41% Associate degree 13% Bachelor’s degree 26% Master’s degree or higher 11%
Chu [8] [2012]	Republic of Korea	291	36.4 ± 15.0	Oncology Cardiology Endocrinology Gastroenterology Immunology Neurology Others	Adult patients	ND	Freedom to withdrawal 78.2% Compensation 43.4% Randomisation 49.8% Fulfilling inclusion criteria to be eligible 82.5% Approval of bioethics committee 78.7% Voluntary participation 53.6%	≤ High school 28.9% ≥ College 71.1%
Chaisson [9] [2011]	Botswana	1835	33	Infectious disease	Adult patients	Within 30 days after ICP	Purpose of the study 90–97% Placebo and blinding 64–65% Voluntary participation 69–77% Risk 87–94% Freedom to withdrawal 76–77%	No formal education 7% Primary school 28% Secondary school 59% Tertiary 9%
Ellis [10] [2010]	USA/Mali	171 89 700	30 (18–50) 27 (18–50) ND	Infectious disease (malaria vaccine)	Adult patients and parents or guardians	Directly after ICP	Freedom to withdrawal 93–98%^1^ Safety and side effects 48–85%^1^	ND
Minnies [6] [2008]	South Africa	192	26 (16–44)	Infectious disease	Parents or guardians	Within 1 h after ICP	Reason to participate 85.4% Purpose of the study 80.6% Procedures 60.9% Duration of the study 66.3% Risks 79.2% Benefits 51.3% Voluntary participation 65.1% Confidentiality 73.3% Storage and use 82.9%	Grade ≤ 6 23.4% Grade 7–11 42.8% Grade ≥ 12 33.9%
Bergenmar [11] [2008]	Sweden	282	60 (32–82)	Oncology	Adult patients	Within max. 2 weeks after ICP	Voluntary participation mean 96.2 (13.2) Confidentiality mean 84.3 (26.2) Potential risks or discomfort mean 76.7 (28.2) Withdrawal criteria > 90% Randomisation > 80% Side effects > 10%	Compulsory school (1–9 years) 24% Senior high school (10–12 years) 29% University education (13–16 years) 38% Higher university education (> 16 years) 9%
Bertoli [12] [2007]	Argentina	105	56.3 ± 11.8	Rheumatology	Adult patients	ND	Participant’s blinding 64.9% Investigator’s blinding 27.3% Freedom to withdrawal 77.8% Randomisation 10% Placebo 47–65.5%^2^ Confidentiality 52%	Illiterate 1.9% Elementary school (7 years of education) 48.6% High school 28.6% Higher educational degrees 19.0%
Krosin [13] [2006]	Mali	163	ND	Infectious disease (malaria vaccine)	Adult patients	Within 2 days after ICP	Voluntary participation 57% Compensation 44% Withdrawal criteria 10% Withdrawal consequences 44% Randomisation and placebo 68% Side effects 7% Lay scientific knowledge 73%	Primary education 67% Literate to some degree 70%
Criscione [14] [2003]	USA	30	44.9 ± 9.8	Rheumatology	Adult patients	7–28 days after ICP	Voluntary participation 77% Freedom to withdrawal 63% Participant’s blinding 53% Investigator’s blinding 80% Randomisation 50% Placebo 87% Risk and safety 30%	Years of education, median 12.5
Pope [15] [2003]	Canada	190	63 (22–84)	Cardiology, ophthalmology, rheumatology	Adult patients	2 months to 5 years after ICP	Participant’s blinding 14–47%^3^ Investigator’s blinding 9–60%^3^ Randomisation 20–66%^3^ Placebo 13–49% ^3^	Elementary school 18% High school 45% Higher education 36%
McNally [16] [2001]	UK	29	32	Infectious disease	Parents or guardians	ND	Randomisation 75% Blinding 75% Placebo 64%	No formal education 18% ≥ Advanced “A” level 21% Ordinary “O” level 39% National vocational qualification 11% Certificate of secondary education 11%
Itoh [17] [1997]	Japan	32	58 (30–68)	Oncology	Adult patients	After ICP before drug treatment	Freedom to withdrawal 97% Right to therapeutic choice 88%	Elementary school 16% High school 47% Higher education 28% Unknown 9%
Harrison [18] [1995]	USA	175	37 (18–56)	Infectious disease (HIV vaccine)	Adult patients	Before ICP	Confidentiality 95–100%^4^ Side effects 83–100%^4^ Randomisation 68–96% ^4^	ND

*ND* no data, *ICP* informed consent process

^1^Depending on population Mali parents/guardians of child participants vs USA adult participants

^2^Depending on the trial

^3^Depending on the treatment study: cardiology, ophthalmology, rheumatology

^4^Depending on subgroup: eligible and ineligible for the vaccine trial, and then further divided into injection drug users (IDU) and others within each group

### Understanding of informed consent

Questionnaires that examined participants’ understanding of IC components included true/false items [9, 10, 18], multiple choice items, and the Quality of Informed Consent survey [7, 11]. Questionnaires differed in the number of items and content. All questionnaires examined participants’ recall of IC content, except one, where participants used the IC text to find the answers to the survey questions [19]. Additionally, the elapsed time between participants’ experience of the informed consent process and their IC research participation ranged from before the actual IC [18] process to 5 years after the IC [15]; four studies did not report this measure [8, 12, 16, 19].

Three studies examined participants’ understanding of the research purpose. Schumacher et al. reported that all participants understood that they were participating in a research study and recognised its purpose [7]. In the remaining two studies, most participants comprehended the study aims (70–90%) [9, 19].

Voluntary participation was examined in seven studies [6–9, 11, 13, 14]. Bergenmar et al. reported the highest level of comprehension, with 96% of participants comprehending the voluntary nature of their participation [11]. In contrast, Chu et al. reported the lowest level of comprehension, with 53.6% [8]. Additionally, Krosin et al. noted a significant difference between urban and rural participants, with 85% and 21%, respectively, showing comprehension of the voluntary nature of participation [13]. Chu et al. reported that 53.6% patients understood that physicians should not persuade them to participate in a study [8]. Criscione et al. reported that 10% of participants indicated that their personal doctor would mind if they dropped out of the study [14].

Freedom to withdraw was reported in eight studies [6–8, 10, 12, 14, 17, 19], which was a relatively well-comprehended IC component, with the lowest level of 63% reported by Criscione et al. [14]. Ponzio et al. reported that all participants correctly understood their right to withdraw at any time [19]. Additionally, one study reported on awareness of withdrawal consequences, with 44% demonstrating comprehension of this point; and withdrawal criteria, with only 10% showing comprehension [13].

Comprehension of randomisation was investigated in seven studies, with Harrison’s study reporting the highest level of understanding (96%), and Bertoli et al. reporting the lowest (10%) [8, 11, 12, 14–16]. Similarly, the understating of placebo and active treatment ranged from 13% [15] to 97% [9]. Differences by specialty regarding comprehension of the placebo concept were noted by Pope et al., with the lowest comprehension reported in the ophthalmology group (13%) and the highest in the rheumatology group (49%) [15].

Risks and benefits were explored in nine [6, 7, 9–11, 13, 14, 18, 19] and three studies [6, 7, 19], respectively. Krosin et al. reported that only 7% of patients comprehended risks associated with involvement in clinical trials [13]. In contrast, in one group, all patients (who could use the IC text to find questionnaire answers) were aware of potential side effects and risks of the treatment [19]. Ponzio et al. reported the highest between-group differences in the comprehension of study benefits, which ranged from 35.5 to 96.6%, depending on whether participants were successful in finding the answer in the IC text [19].

It is worth noting that Schumacher et al. reported that patients were not aware that the proposed treatment was experimental and not standard therapy [7]. Additionally, only 20% of participants understood that the benefits of treatment were uncertain and that participation was associated with additional risks. Similarly, over 30% of patients were not aware that alternative treatments were available.

Furthermore, Chu et al. found that only 43.4% of patients understood that they would not be reimbursed for all adverse events related to the study. Of note, the authors did not specify the conditions regarding reimbursement. The number of correct responses was higher in the healthy control group than in the patient group (excluding the last question related to reimbursement) [8].

Finally, Bertoli et al. reported that 86 participants (83.5%) recalled that they had fully read the informed consent form, while 11.7% had partially read it and 9% did not remember to what extent they had read it. Interestingly, most patients (51.4%) rated their knowledge about the study as high, but objective evaluation of participants’ knowledge showed that only 14.3% demonstrated a high level of knowledge, and 58.1% and 27.6% showed intermediate and low knowledge, respectively [12]. Pope et al. reported that 18% of participants admitted that they had not fully read the study information letter and 10% admitted that they were afraid to ask questions [15].

### Assessment of risk of bias

Studies included in this review were either randomised nor blinded for the outcomes related to IC. Therefore, the assessment of risk of bias was not possible [20].

## Discussion

We concluded that there are significant discrepancies in research participants’ understanding of voluntary participation, blinding, and freedom to withdraw. Only rarely did all participants respond correctly to questionnaire items, indicating that they actually comprehended what they consented to. We found that participants presented the highest level of understanding (over 50%) about voluntary participation, blinding (excluding knowledge about investigators’ blinding), and freedom to withdraw at any time. Further, our results suggest that only a small minority of patients had a clear and accurate understanding of all aspects of their consent. In particular, patients presented significant difficulties in grasping the concept of placebo randomisation, safety, risks, and side effects [7–16, 18, 19]. Additionally, some patients had very limited comprehension of the research benefits [6, 19].

Our findings are consistent with the results of previous meta-analyses on the quality of the informed consent process in clinical trials [21]. However, in general, patients included in our review demonstrated lower levels of comprehension. Tam et al. [21] reported that two-thirds of participants (the highest reported level) understood the freedom to withdraw from a study at any time, followed by the nature of the study, the voluntary nature of participation, and the potential benefits. In contrast, our results showed that 69.6% of participants understood the purpose of the study and only 54.9% could name at least one risk. Finally, approximately half of the participants understood placebo and randomisation concepts. However, in contrast to our review, Tam et al. included data from interviews in their analysis. Surprisingly, in 58.5% of interviews, participants could not establish whether the interviewers were investigators in the original clinical trial and, as such, could influence the results [21]. In a previous systematic review of clinical trial IC or surgery IC, Falagas et al. concluded that only 50% of participants properly understood all IC components [22].

These findings demonstrate that crucial information, including risks and benefits, voluntariness, and the relation of trials to standard therapy, are not actually comprehended by a substantial number of participants. This seriously undermines the present practice of providing a sound ethical basis for experimenting with human subjects. Moreover, it seems that patients’ understanding of specific IC components has not changed over the past 20 years [21]. The guidelines for good clinical practice in trials, introduced by the World Health Organization 20 years ago, have not affected patients’ comprehension [21, 23].

It is natural to expect a correlation between general health literacy and comprehension of information relevant to informed consent. The extent to which deficits in understanding consent depend on insufficient general health literacy remains to be examined. However, this may not be crucial to the existing practice viability in obtaining informed consent as a safeguard for respecting patients’ autonomy in clinical trials. It is inevitable that patients recruited for clinical trials will have varying education and health literacy levels. There is no reason to assume that patients included in clinical trials have lower than average health literacy. Therefore, the outcomes suggest that, in the daily practice of clinical trials, patients with diverse education and health literacy levels agree to participate in medical testing based on defective, or at least incomplete, comprehension of the relevant information. This suggests that the present routines regarding patients’ autonomy (and thus—dignity) in clinical trials are ethically questionable, if not explicitly flawed.

None of the studies included in our review directly examined relations between patients’ health literacy and their level of comprehension regarding the consent they granted. However, Chaisson et al. reported that patients’ education was considered as a factor potentially influencing their understanding of the consent. They administered questionnaires in English or Setswana and concluded that participants who had a higher education level or chose to complete the questionnaires in English rather than Setswana demonstrated better overall comprehension. Similarly, Schumacher et al. and Krosin et al. found significant correlations between comprehension scores and formal education levels [7, 13]. Ellis et al. distributed a survey to adult participants in the USA and Mali, plus the parents or guardians of a child in an additional group of Mali participants. Within Malian adults, only 9% signed the IC, while the remaining 91% provided a fingerprint. In the Malian parents or guardians group, 84% provided a fingerprint rather than a signature. The literacy rate for parents or guardians varied between sites, ranging from 3 to 17%. Of note, the questionnaire was initially intended to teach participants rather than to collect data [10]. The researchers found that patients’ literacy was not a significant factor in their ability to understand the consent they were asked to grant.

The studies included in this review have some limitations that should be considered while interpreting the results. First, they demonstrated a high level of heterogeneity in sample size and type of underlying medical condition. We speculate that this may also have influenced patients’ understanding of informed consent. For example, Pope et al. reported significant differences in the level of understanding within patients recruited to rheumatology, ophthalmology, and cardiology studies [15]. Similarly, Chu et al. reported that 61% of healthy controls were recruited from phase I trials, in which they were under the close monitoring and care of the researchers, while 80.8% of patients were recruited from phase III or IV trials that were conducted in outpatient clinics [8]. Second, especially in oncology, the presumed potential benefit of a novel treatment may exceed the presumed risk of the study, biasing patients towards consenting to participate in the trial despite a limited understanding of its experimental nature. Third, although education level was usually mentioned, health literacy was not examined in most studies. Additionally, other factors related to underlying disease may influence patients’ comprehension, including fatigue, depression, cognitive status, and emotional factors associated with study inclusion and doctor’s office visits. The resulting scope of interference with patients’ ability to grasp the full meaning of the consent remains unexamined. However, such factors should be considered when assessing the current practice of obtaining informed consent. Finally, the scales used to examine patients’ understanding differed across studies and ranged from multiple choice to Likert-like scale items, which limited our ability to adequately compare patients’ understanding across studies.

In many cases, patients may be unaware that they lack understanding and therefore do not ask for clarification. In some cases, the information on expected therapeutic benefits may overshadow other aspects of the project, making patients less receptive to technical or more discouraging sides of the trial. Interestingly, our findings suggest that mothers asked to consent to including their children in a clinical trial were more determined to comprehend all relevant information than adult patients deciding on their own involvement in a trial. However, this is based on the results from a single study in this review [6].

The relatively consistent series of empirical findings opens further questions that have not been satisfactorily addressed in the literature to date. We hypothesise that patients seriously overrate their own level of comprehension. The extent to which they mistakenly feel that they have understood all relevant information while, in fact, they miss many important aspects of the consent present an interesting question that we are preparing to investigate. Similarly, physicians may seriously overrate their patients’ level of comprehension based on (1) their own efforts to effectuate patients’ understanding, (2) their confidence in patients’ declaration of understanding and satisfaction, and (3) their own health literacy influencing their belief that the information offered to patients is easier to understand than is actually the case.

Thus, further research should target empirical testing of the hypothesised discrepancies between (1) the actual level of understanding by patients regarding what they consented to, (2) their subjective confidence that they understood what they consented to, and (3) physicians’ confidence that their patients actually understood what they consented to.

Noting the scarcity of analogous research on the actual understanding of consent by patients in regular therapeutic practice, we recommend that future studies examine such comprehension in ordinary medical settings rather than only through the context of clinical trials. Since physicians typically take more care and effort to explain all relevant aspects of a clinical trial, we assume that, in a standard therapeutic setting, lack of comprehension regarding consent may be even larger. Therefore, a lack of relevant research in therapeutic clinical settings constitutes a remarkable gap in a crucial aspect of ethically viable medical practice.

## Conclusion

We found that the level of comprehension regarding informed consent components, such as voluntary participation, blinding, and freedom to withdraw, was low, being understood by only half of the patients. This seriously undermines the ethical foundations of current practices for obtaining consent in clinical trials, potentially also challenging the standard approach to safeguarding patients’ autonomy in ordinary medical settings.

## Data Availability

The datasets used and/or analysed during the current study are available from the corresponding author on reasonable request.

## Abbreviations

IC: Informed consent
IDU: Injection drug users
MEDLINE: Medical Literature analysis and retrieval system online (a database)
MeSH: Medical subject headings
Embase: Excerpta Medica database
